# The CXCL10/CXCR3 Axis and Cardiac Inflammation: Implications for Immunotherapy to Treat Infectious and Noninfectious Diseases of the Heart

**DOI:** 10.1155/2016/4396368

**Published:** 2016-10-03

**Authors:** Raffaele Altara, Ziad Mallat, George W. Booz, Fouad A. Zouein

**Affiliations:** ^1^Department of Pharmacology and Toxicology, School of Medicine, University of Mississippi Medical Center, Jackson, MS 39216-4500, USA; ^2^Division of Cardiovascular Medicine, Department of Medicine, University of Cambridge, Cambridge CB20 SZ, UK; ^3^Institut National de la Santé et de la Recherche Médicale (Inserm), Unit 970, Paris Cardiovascular Research Center, 75015 Paris, France; ^4^Department of Pharmacology and Toxicology, American University of Beirut, Faculty of Medicine, Beirut 1107 2020, Lebanon

## Abstract

Accumulating evidence reveals involvement of T lymphocytes and adaptive immunity in the chronic inflammation associated with infectious and noninfectious diseases of the heart, including coronary artery disease, Kawasaki disease, myocarditis, dilated cardiomyopathies, Chagas, hypertensive left ventricular (LV) hypertrophy, and nonischemic heart failure. Chemokine CXCL10 is elevated in cardiovascular diseases, along with increased cardiac infiltration of proinflammatory Th1 and cytotoxic T cells. CXCL10 is a chemoattractant for these T cells and polarizing factor for the proinflammatory phenotype. Thus, targeting the CXCL10 receptor CXCR3 is a promising therapeutic approach to treating cardiac inflammation. Due to biased signaling CXCR3 also couples to anti-inflammatory signaling and immunosuppressive regulatory T cell formation when activated by CXCL11. Numbers and functionality of regulatory T cells are reduced in patients with cardiac inflammation, supporting the utility of biased agonists or biologicals to simultaneously block the pro-inflammatory and activate the anti-inflammatory actions of CXCR3. Other immunotherapy strategies to boost regulatory T cell actions include intravenous immunoglobulin (IVIG) therapy, adoptive transfer, immunoadsorption, and low-dose interleukin-2/interleukin-2 antibody complexes. Pharmacological approaches include sphingosine 1-phosphate receptor 1 agonists and vitamin D supplementation. A combined strategy of switching CXCR3 signaling from pro- to anti-inflammatory and improving Treg functionality is predicted to synergistically lessen adverse cardiac remodeling.

## 1. Introduction

The chemokine receptor CXCR3 is a Class A seven-transmembrane-domain or G protein-coupled receptor (GPCR) that is involved primarily in chemotaxis of certain immune cells, inhibition of angiogenesis, and Th1 cell polarization [1–3]. CXCR3 is expressed by various effector T lymphocytes, including CD4^+^ T helper 1 (Th1) cells, CD8^+^ cytotoxic T lymphocytes (CTL), and CD4^+^ and CD8^+^ memory T cells, as well as monocytes, M1 macrophages, natural killer (NK) cells, subsets of B-cells, mast cells, endothelial cells, and vascular smooth muscle cells [1–4]. CXCR3 couples to G*α*_i_ protein [5, 6] and although not extensively studied, it has been shown to activate a number of signaling pathways that are generally associated with GPCRs such as increases in intracellular calcium and activation of MAP kinases and PI3K/Akt signaling [4, 7, 8]. The principal agonists of CXCR3 are CXCL9 (Mig), CXCL10 (IP-10), and CXCL11 (I-TAC). The human equivalent of the murine form of CXCR3 is CXCR3A and unless noted otherwise CXCR3 is used in this review to include both murine and human isoforms. Two additional splice variants of CXCR3 are expressed in humans, CXCR3B and CXCR3-alt. CXCR3B, which couples to G_s_, is the receptor isoform expressed in microvascular endothelial cells and is linked to inhibition of angiogenesis and induction of apoptosis [2, 3]. Besides CXCL9, CXCL10, and CXCL11, CXCR3B and CXCR3 are activated by CXCL4 and CXCL4L1, chemokines that are released by platelets and have been implicated in atherogenesis and acute coronary syndrome [3, 9, 10]. CXCR3-alt is a truncated form of CXCR3 that is selectively activated by CXCL11 [2–4].

CXCR3 is associated with the pathophysiology of Th1-type diseases, including infections of various etiologies and autoimmune disorders [1, 3]. Although CXCR3 is activated by CXCL9, CXCL10, and CXCL11, the outcome is different with growing evidence that CXCL9 and CXCL10 are essentially proinflammatory, while CXCL11 has anti-inflammatory actions [11, 12]. Over the last decade, numerous studies have documented elevated circulating levels of CXCL10 in wide-ranging infectious and autoimmune diseases, autoimmune encephalomyelitis, Crohn's disease, tuberculosis, thyroid autoimmune diseases, and type 1 diabetes, as well as several cancers [13–18]. Recent evidence from us and others [1] has revealed the importance of the CXCL10/CXCR3 axis in cardiovascular diseases. As discussed elsewhere [11], CXCL9 and CXCL10 are two of only 8–10 chemokines that are sufficient to sustain an inflammatory response. In addition, the homing signature for memory T cells to the heart from mediastinal lymph nodes is c-Met^+^CCR4^+^CXCR3^+^. While c-Met triggering supports cardiotropic T cell recirculation, CXCR3 and CCR4 engagement* via* tissue-released CXCL10 and CCL4, respectively, sustains recruitment in heart inflammation [19, 20]. In this review, we present an overview of the role of CXCL9 and CXCL10 in infectious and noninfectious diseases of the heart and its implications for immunotherapy.

## 2. CXCR3 Biased Signaling

Recently, Zohar et al. [21] showed that CXCL9 and CXCL10 drive effector Th1/Th17 cell polarization via STAT1, STAT4, and STAT5 activation, thereby promoting inflammation. In contrast, CXCL11, which exhibits relatively higher binding affinity for CXCR3, drives development of FOXP3 (forkhead box P3)-negative IL-10^high^ T regulatory 1 (Tr1) cells and IL-4^hi^ Th2 cells* via* STAT3 and STAT6 activation and was demonstrated to dampen inflammation [21]. The opposite actions of the CXCR3 agonists are likely the consequence of the biased signaling that is a fixture of GPCRs, which can activate both G protein-dependent and protein-independent signaling cascades, the latter occurring * via* *β*-arrestin 2 recruitment [1, 11, 12]. Biased allosteric agonists of CXCR3 that selectively activate *β*-arrestin or G protein-dependent signaling are in development and may have utility in immunotherapy [22].

## 3. Coronary Artery Disease (Ischemic Heart Disease)

Coronary artery disease (CAD), which progresses to coronary heart disease, is a leading cause of death in the USA and globally [23, 24]. CAD is caused by atherosclerosis within the arteries of the heart, a chronic inflammatory condition associated with waxy plaque buildup [25]. CXCR3 expressing monocytes/macrophages, Th1 cells, NK cells, and CTL cells play a critical role in atheromatous plaque progression and eventual disruption [1]. Ruptured or ulcerated plaques cause formation of a thrombus that precipitates an acute coronary syndrome, such as unstable angina or a heart attack.

Endothelial dysfunction, increased vascular permeability, increased expression of adhesion molecules on endothelial cells for leukocytes, and increased plasma levels of low density lipoprotein (LDL) are initiating factors in atherosclerosis [1]. LDL, which accumulates in the intima, undergoes oxidation by macrophages and endothelial cells, as well as by VSMC that migrate into the intima from the media and proliferate. In response to the oxidized LDL and plasma LDL, endothelial cells secrete proinflammatory cytokines and chemokines (MCP-1/CCL2, fractalkine, and CXCR3 ligands) that attract monocytes, which differentiate into dendritic cells or macrophages that accumulate oxidized LDL to become foam cells. T cells are recruited into the intima, and dendritic and NK cells help induce the CD4^+^ Th1 phenotype, which is the most abundant T cell population in human atherosclerotic plaques [26]. CXCR3 is required for optimal Th1 generation [27]. Th1 cells, as do NK cells, produce IFN-*γ*, which contributes to Th1 polarization, activates proinflammatory M1 macrophages, and induces apoptosis. The atheromatous plaque that builds up in the artery wall is made up of an accumulation of lipids, fibrous connective tissue, macrophages, and cellular debris that arises from the cytolytic actions of oxidized LDL, NK cells, IFN-*γ*, and CTL cells on macrophages, foam cells, VSMC, and endothelial cells. A fibrous coat of extracellular matrix proteins produced by VSMC stabilizes the plaque, but proinflammatory M1 macrophages secrete metalloproteinases in response to IFN-*γ* that degrade the fibrous cap and enhance its vulnerability to rupture.

CXCL10 is reported to be expressed by endothelial cells, smooth muscle cells, and macrophages during the formation of atherosclerotic lesions in both preclinical and clinical studies [28, 29]. Suppression of CXCL10 bioactivity in Apo-E deficient mice resulted in a more stable plaque phenotype with less macrophage activation, along with more smooth muscle cells and collagen abundance [30]. The mechanistic role of CXCL10 in the pathogenesis of atherosclerotic plaque growth and destabilization is not yet resolved. Of note, CXCL10 concentrations increase in patients with a more vulnerable plaque phenotype [30]. Unstable plaques have increased levels of Th1, NK, and CTL cells and decreased levels of anti-inflammatory regulatory T (Treg) cells [31]. Recent studies show that the relative levels of Treg cells are reduced and their functionality is impaired in patients with CAD [32, 33]. Knockout of CXCL10 in the apolipoprotein E-deficient mouse model of atherosclerosis was associated with increased Treg cell numbers and activity, along with a reduction in lesion formation [34].

Circulating levels of CXCL10 are elevated in patients with coronary artery disease [35, 36]. Notably, CXCL10 was also reported to be produced by the endothelium of mouse coronary blood vessels infused with angiotensin II [37], human coronary artery endothelial cells treated with TNF-*α* [38], and rat cardiac microvascular endothelial cells subjected to hypoxia/ischemia [39]. Patients with acute myocardial infarction (AMI) showed significantly higher serum levels of CXCL10 than control subjects and patients with stable angina pectoris [40]. Although serum CXCL10 levels were negatively correlated with infarct size, these results in terms of pathogenic implications and determining cause* versus* effect relationships have limitations. First, during AMI there is a massive systemic inflammatory insult in which CXCL10 levels are expected to be high. It would be interesting to test blood concentration of CXCL10 within the first 3 hours after angina onset during AMI when systemic activation is not yet started. Secondly, the pathogenic mechanisms of plaque rupture may involve factors acting locally without necessarily showing a high systemic blood concentration. It would be interesting to analyze CXCL10 in samples of blood obtained by thrombus-aspiration during coronary artery percutaneous intervention (PCI) in patients with unstable coronary artery disease. In patients with first-time ST-segment elevation AMI, high circulating levels of CCL4, CXCL16, CXCL8, and CXCL10 within the first week after PCI were found to be positively correlated with the degree of myocardial damage [41].

## 4. Kawasaki Disease

Kawasaki disease is an autoimmune disease that manifests as a systemic vasculitis with a predilection for coronary arteries [42]. The disease occurs in children under 5 years of age and a preexisting viral infection may have a role in its development. During the acute phase of Kawasaki disease the immune system is highly activated and includes both Th1 and Th2 subsets [43]. Recently, Ko et al. [44] reported that CXCL10 is a good biomarker/predictor of Kawasaki disease and, furthermore, that CXCR3 is activated in the T cells of patients with acute Kawasaki disease. In addition, several studies report that numbers and functionality of Treg cells are reduced in Kawasaki patients [45–48].

## 5. Myocarditis and Chagas Heart Disease

Myocarditis or inflammation of the myocardium is a heterogeneous group of disorders initiated by various pathogens, including worms, bacteria, protozoa, rickettsia, and most commonly viruses [49]. Myocarditis may lead to heart failure and sudden death. Autoimmunity after viral myocarditis is thought to cause dilated cardiomyopathy, which is characterized by ventricular dilation and contractile dysfunction [50, 51].

CXCL10 is elevated in the heart following viral and nonviral infection and has the characteristics of a biomarker in rodent models of myocarditis [52, 53]. Yue et al. [54] reported findings showing that CXCL10 contributes to the pathogenesis of viral myocarditis. In their study, myocarditis was induced with Coxsackievirus B3 (CVB3), the primary cause of viral myocarditis, in mice that overexpressed a CXCL10 mutant protein without functional activity in order to antagonize endogenous CXCL10. These mice exhibited ameliorated disease progression, including reduced cardiac thickening (due to inflammatory edema), inflammation, and cell death, as well as improved survival when compared to wild-type mice [54]. The authors concluded that CXCL10 plays a crucial role in recruitment of Th1 cells to the heart, leading to the increase in detrimental proinflammatory Th1 cytokines. These findings have implications for interferon treatment, which is beneficial for some forms of viral myocarditis [55]. Following CVB3 infection, the rise in IFN-*γ* stimulates CXCL10 expression in cardiac myocytes and other cardiac cells. Yuan et al. [56] reported that CXCL10 inhibits CVB3 replication at early stage of infection, consequently protecting cardiac myocytes from damage and improving heart function. This antiviral activity of CXCL10 entails the regulation of natural killer (NK) cell infiltration into the myocardium and associated IFN-*γ* expression. However, the transient antiviral effect of CXCL10 was shown to be insufficient for viral clearance and in preventing death during acute inflammation stages in their mouse model. Other chemokines or cytokines were proposed to play an important role in clearance of viruses. No simple explanation seems to explain the disparate findings of Yuan et al. [56] and Yue et al. [54] on whether myocardial CXCL10 is harmful or beneficial in the context of acute CVB3 myocarditis, although timing and dosage levels of CXCL10 and effective viral clearance* versus* Th1 recruitment are likely contributing factors.

Evidence indicates the major contribution of autoimmunity to the etiology of myocarditis and thus adoptive transfer of Tregs and/or stimulating their differentiation are promising therapeutic approaches [49, 50]. Another possible immunotherapy approach is humanized monoclonal antibodies targeting IL-17 derived from Th17 cells [49]. Both Th1 and Th17 cells drive myocarditis, with Th17 cells playing an important part in the development of dilated cardiomyopathy [57].

Chagas disease is a tropical disease that results from infection with the protozoan parasite* Trypanosoma cruzi* and affects ~10 million individuals worldwide but is most prevalent in Latin America [58, 59]. In some, 20–30%, of infected individuals, chronic infection leads to a potentially fatal cardiomyopathy known as Chagas heart disease, generally 10–20 years after the initial infection [60, 61]. Chagas heart disease is characterized by marked inflammation and fibrosis of the heart, along with cardiac edema, myofibrillar destruction, chamber dilation, and loss of contractile function. The etiology of Chagas heart disease is not fully understood and likely multifactorial, with a contribution of an autoimmune response due in part to molecular mimicry between antigenic determinants of* T. cruzi* and human (cardiac) antigens [62, 63]. With heart failure in Chagas disease, myocardial levels of CD8^+^ and CD4^+^ T cells are increased, with a predominance of CD8^+^ T cells [61, 64, 65]. In addition, the myocardium exhibits a strong Th1 cytokine profile with increased expression of IFN-*γ* and IL-18 genes that correlate with ventricular dilation [64, 65]. Circulating levels of IFN-*γ* are elevated during chronic Chagas disease [61] and were reported to be inversely correlated to left ventricular ejection fraction (LVEF) [66]. In contrast, Chagas-related heart failure is associated with reduced myocardial levels of Treg cells [61, 64], and circulating Treg activity was reported to be reduced in moderate or severe cardiomyopathy with activity directly correlated to LVEF [66].

Several studies have implicated CXCL10 in Chagas cardiomyopathy. Increased plasma levels of CXCL10 were detected in patients with chronic Chagas disease [67], and LV mRNA expression levels of* CXCL10* were found to be elevated in patients with Chagas cardiomyopathy [68]. Recently, evidence was provided that polymorphisms in the* CXCL9* and* CXCL10* genes controlled the expression of chemokines in the myocardium and the degree of myocarditis in Chagas cardiomyopathy [69].

Behçet's disease is an autoimmune or autoinflammatory disorder common in the Middle East, Asia, and Japan. The basis for the pathogenesis of Behçet's disease is not known, although a number of factors have been proposed to have a role, including viral, bacterial, environmental, genetic, and immune factors. Behçet's is caused by small-vessel systemic vasculitis that very often affects the heart in diverse ways, including endomyocardial fibrosis, intracardiac thrombus, endocarditis, pericarditis, myocarditis, coronary arteritis, myocardial infarction, and valvular disease [70]. The cardiomyopathy may be ischemic, nonischemic, or inflammatory in nature and may manifest as asymptomatic systolic or diastolic dysfunction or overt systolic or diastolic heart failure [70]. Recently, monocytes of Behçet's patients were found to have dysfunctional posttranscriptional regulation of CXCL10 mRNA that resulted in overexpression of CXCL10 protein with IFN-*γ* stimulation [70]. Thus, overexpression of CXCL10 may contribute to the pathogenesis of Behçet's disease. In general, the role of CXCL10 in immune-mediated and autoimmune myocarditis is little studied; however, based on studies of infective myocarditis, a critical role for CXCL10 is likely. The role of CXCL10 in cardiac allograft transplantation rejection is discussed elsewhere [1].

## 6. LV Hypertrophy and Nonischemic Heart Failure

After CAD, hypertension is the most common risk factor for heart failure and accounts for ~25% of heart failure cases [71]. In the elderly, as many as 68% of heart failure cases are linked to hypertension and community-based studies indicate that hypertension contributes to heart failure in 60% of patients [72]. Hypertension causes a number of adverse remodeling events at the cellular and tissue level of the heart, including cardiac myocyte hypertrophy and gene reprograming, activation of cardiac fibroblasts, interstitial and perivascular fibrosis, and capillary refraction [73–75]. These alterations ultimately cause marked changes in the overall geometry of the heart that may progress to heart failure and the inability of the heart to adequately meet the oxygen and energy demands of the body. Heart failure (HF) may manifest clinically with either preserved or reduced LVEF, which are designated HFpEF (so-called diastolic heart failure) and HFrEF (systolic heart failure), respectively [75].

Hypertension results in concentric LV hypertrophy, which may progress to ventricular dilation and eventual HFrEF because of poorly understood means that may include ischemic injury [76, 77]. Related to this, volume overload due to fluid retention and impaired kidney function may cause a dilated pattern of eccentric LV hypertrophy with hypertension that leads to HFrEF [77]. Generally, cardiac remodeling with hypertension reflects a combination of both concentric and eccentric patterns of remodeling [77]. Concentric hypertrophy is also a characteristic of HFpEF, which typically has hypertension as the major comorbidity [78]. In addition, microvascular dysfunction concomitant to hypertension is thought to be a contributing factor for HFpEF [79]. The relative importance of CXCL10 in concentric* versus* eccentric LV hypertrophy, as well as their progression to heart failure, is not known.

Numerous preclinical and clinical studies have implicated marked activation of neurohormonal drive to the heart in the pathoetiology of LV hypertrophy and its progression to heart failure [80]. Neurohormonal drive, namely, activation of the sympathetic and renin-angiotensin-aldosterone systems, is generally thought to directly cause adverse remodeling of the heart. At the same time, there is evidence for indirect actions of neurohormonal stimulation on cardiac remodeling* via* activation of innate immunity and inflammation, especially the induction of heart-derived proinflammatory cytokines [81]. In chronic heart failure, an increased Th1/Th2 ratio is seen [82], but whether increased Th1 cell levels contribute to heart failure progression or simply are a consequence of heart failure is unresolved.

Exciting new findings have now implicated adaptive immunity and T cells more directly as causal agents in hypertensive LV hypertrophy and resultant heart failure by poorly understood means. These preclinical studies employed the mouse model of transverse aortic constriction- (TAC-) induced heart failure to mimic the impact of high blood pressure on the heart. We observed that circulating levels of CXCL9 and CXCL10 are elevated in TAC mice [83]. Laroumanie et al. [84] reported increased recruitment of activated CD4^+^ and CD8^+^ T cells and elevated levels of several chemokines for T cells and monocytes, including CXCL10, in ventricular tissues from mice with TAC-induced heart failure. TAC-induced ventricular dilation and fibrosis was prevented and contractile dysfunction was attenuated in mice deficient in mature B and T lymphocytes due to knockout of RAG2, although cardiac hypertrophy was still observed. T cell replenishment in RAG2 knockout mice restored the TAC-induced heart failure phenotype. In addition, elimination of CD4^+^ T cells (MHCII knockout) but not CD8^+^ T cells (CD8^+^ knockout) prevented TAC-induced cardiac fibrosis and failure, suggesting a critical involvement of T helper cells. This conclusion was further supported by the observation that mice with transgenic T cell receptor specific for ovalbumin did not develop heart failure and fibrosis with TAC. Altogether these findings suggest that activation of CD4^+^ T cells in hypertension causes interstitial and perivascular fibrosis that leads to functional and morphological changes in the heart conducive to the development of heart failure. However, it should be noted that an earlier study reported that coronary vessels of RAG1 knockout mice exhibited more intimal hyperplasia and perivascular fibrosis compared to wild-type mice following TAC [85]. The basis for the discrepant findings of the two studies is not clear. More recently, Nevers et al. [86] also investigated the role of T cells in cardiac remodeling in response to TAC-induced pressure overload. They observed that the development of systolic dysfunction was associated with the kinetics of T cell infiltration into the left ventricle and evidence was provided that most of the infiltrating T cells were IFN-*γ* secreting Th1 cells. LV systolic and diastolic function were preserved with TAC in T cell deficient mice (T cell receptor (TCR) knockout), and LV hypertrophy, fibrosis, and inflammation were markedly attenuated. In addition, T cell depletion with an anti-CD3 antibody prevented heart failure in wild-type mice. Unresolved at present is the identity of the antigen(s) responsible for T cell activation in LV hypertrophy and heart failure, and the potential contribution played by the loss of regulatory mechanisms that normally protect the heart from T cells [87].

In contrast to the involvement of adaptive immunity in TAC, Ma et al. [88] provided evidence that CD8^+^ T cells play a critical role in perivascular and interstitial fibrosis in the angiotensin II infusion model of hypertensive cardiac remodeling through the recruitment and activation of macrophages. They found that CD8^+^ T cells are recruited to the heart and activated by IFN-*γ* secreting myocardial cells; recruited macrophages in turn are activated by CD8^+^ T cells in contact-dependent, but TCR-independent means. A possible contribution of CD4^+^ T cells to the actions of CD8^+^ T cells will need to be explored.

Circulating levels of CXCL10 are elevated in patients with untreated essential hypertension [89]. In a small cohort, we observed that the CXCR3 chemokines, including CXCL10, were present in elevated concentrations in the plasma of patients with symptomatic diastolic LV dysfunction indicative of HFpEF or early stage HFrEF [90]. The magnitude of their increase was independent of the extent of hypertension and the CXCR3 agonists enhanced diagnostic accuracy over and beyond NT-pro BNP. More recently, we reported that circulating CXCL10, MIP-1*α*, and CD40 ligand were the best indicators for differentiating healthy and heart failure subjects [91]. We found that serum CXCL10 levels were increased in patients with symptomatic heart failure as indexed by NYHA classification II through IV and were positively correlated with serum levels of Th1 proinflammatory cytokines. The findings of these two studies are consistent with the idea that inflammation is involved in the pathogenesis of heart failure with CXCL10 playing a central role.

Numerous preclinical studies and recent genome-wide association studies (GWAS) support a role for both cytotoxic (CD8^+^) T cells and Th (CD4^+^) lymphocytes in human hypertension [92, 93]. However, accumulating evidence from experimental studies indicates that increasing Treg cell levels in hypertension is an effective strategy to preserve cardiac function, attenuate cardiac hypertrophy and fibrosis, and prevent heart failure progression, independent of any blood pressure lowering effects [94–96]. Reduced circulating levels of Treg cells in heart failure patients have been reported in several studies [97–99].

The role of CXCL10 in other forms of nonischemic heart failure with reduced ejection fraction, such as restrictive cardiomyopathy, ion channelopathies, and diabetic cardiomyopathy, awaits investigation. Recently, Di Luigi et al. [100] reported that the phosphodiesterase type 5 inhibitor sildenafil decreased elevated circulating CXCL10 levels in subjects with diabetic cardiomyopathy, suggesting that sildenafil could be used pharmacologically to mitigate CXCL10-associated inflammation in diabetic cardiomyopathy. Recent findings support a role for CXCL10 in right ventricular (RV) remodeling as well. Waehre et al. [101] found that several chemokines, most notably CXCL10, are upregulated in the pressure-overloaded right ventricle and play a role in myocardial extracellular matrix remodeling in an animal model of pulmonary stenosis. CXCL10 is implicated also in RV dysfunction and inflammation following experimental pulmonary embolism in rats [102].

## 7. Implications for Immunotherapy

The chemokine receptor CXCR3 and its agonist CXCL10 are potential drug targets to treat various cardiovascular diseases.

Potential immunotherapies for cardiac inflammation are as follows:

*Treg Stimulation*

- *IL-2/anti-IL-2* complex treatment to enhance Treg number and activity
- *Targeted cytokine-infused nanoparticles* to stabilize and expand Tregs* in vivo*
- Intravenous immunoglobulin (IVIG) therapy to boost Treg activity
- *Vitamin D* to modulate formation and activity of Tregs
- *Atorvastatin* to enhance Treg number and activity
- *FTY720* to increase Treg levels and activity
- Adoptive Treg cell transfer

*Immunosuppression*

- *Immunoadsorption* to remove circulating antibodies and boost Treg activity
- Phosphodiesterase type 5 inhibitor to decrease CXCL10 formation
- *CXCL11* to stimulate biased GPCR anti-inflammatory signaling
- *PPAR-γ agonists* to block CXCL9, CXCL10, and CXCL11 formation

Levels of CXCL10 are generally elevated with chronic cardiac inflammation, which is associated with enhanced Th1 polarization and infiltration into the myocardium. CXCR3 plays a key role in recruiting various leukocytes to the heart, including monocytes, effector lymphocytes, and CTL cells [1]. Peroxisome proliferator-activated receptor- (PPAR-) *γ* agonists may be a potential pharmacological treatment to block CXCL9, CXCL10, and CXCL11 formation in patients, as PPAR-*γ* agonists show a strong inhibitory effect on their expression and production* in vitro* [103]. Pioglitazone, which lacks the adverse cardiovascular effects of older thiazolidinediones and may be cardiovascular protective, looks promising in this regard, although pioglitazone is contraindicated in heart failure patients likely due to fluid retention [104–107]. Another potential therapeutic approach is the phosphodiesterase type 5 inhibitor sildenafil, which was recently reported to decrease CXCL10 gene expression and protein secretion in human cardiac myocytes and decrease circulating CXCL10 in subjects with diabetic cardiomyopathy [100]. There is substantial evidence that sildenafil has protective effects against adverse remodeling of the heart [108].

Although CXCL9, CXCL10, and CXCL11 all bind to CXCR3, there is evidence that these agonists activate opposing responses due to biased signaling that is a fixture of G protein-coupled receptors [1, 11, 12]. Whereas CXCL9/CXCL10/CXCR3 interactions drive effector Th1 polarization, CXCL11/CXCR3 binding seems to induce an immunotolerant state characterized by T lymphocyte polarization into regulatory Tr1 lymphocytes that produce anti-inflammatory IL-10 [11, 12]. Therefore, inhibiting CXCR3 may not yield definitive findings. Biased agonists have been developed that exert CXCL11-like actions at CXCR3 [22] but have not as yet been assessed in experimental models of cardiovascular diseases. An alternative approach might involve a biological compound. CXCL11 has a short half-life* in vivo*. To address this shortcoming, Zohar et al. [21] generated a stabilized form of CXCL11 by creating a fusion protein in which CXCL11 was linked to IgG1. When administered during ongoing autoimmune encephalomyelitis, the fusion protein suppressed the disease by increasing the number of IL-10-secreting Tr1-like cells (direct effect) and reducing Th1 polarization.

Targeting CXCR3 might be more effective in treating chronic heart inflammation in combination with approaches to enhance Treg numbers or activity, which are generally reduced in cardiovascular diseases (Table 1). Regulatory T cells are immunosuppressive and anti-inflammatory, and a growing number of experimental studies have shown their beneficial effects on the heart in various experimental models of coronary artery disease [109, 110], Kawasaki disease [111], myocarditis and dilated cardiomyopathies [112–117], Chagas heart disease [118, 119], hypertensive LV hypertrophy [95, 96], and nonischemic heart failure [94, 109, 120]. Although not discussed here, boosting Treg numbers or activity in the heart is reported to be beneficial as well in experimental models of infarction-driven remodeling [121–124]. Increasing levels of IL-10-secreting Treg/Tr1 cells may be particularly advantageous, as IL-10 has anti-inflammatory and protective actions on the vasculature and heart [125–132]. Intravenous immunoglobulin (IVIG) therapy has been shown to be effective in treating acute Kawasaki disease in 80–90% of Kawasaki patients with rapid resolution of clinical symptoms and reduced risk of coronary disease [133]. Although the exact basis for the effectiveness of IVIG therapy is not clear, IVIG therapy is thought to modulate the inflammatory process and recent evidence indicates that IVIG therapy acts in part by stimulating an immature myeloid population of dendritic cells that secretes IL-10 and favors expansion of Fc-specific natural Treg cells [134]. IVIG may have promise for treating viral myocarditis [55] and might be effective in treating Chagas [135], as well as chronic heart failure, including both ischemic and idiopathic dilated cardiomyopathies [136].

Adoptive transfer of Tregs and/or stimulating their differentiation are promising therapeutic approaches to target cardiac inflammation [49, 50], although the concerns that must be overcome to make adaptive transfer routine therapy in humans are considerable [137]. However, the safety and efficacy of Treg immunotherapy in humans is supported by preliminary clinical trials for treating graft versus host disease [137, 138]. Immunoadsorption, which is a promising approach for treating myocarditis and dilated cardiomyopathy, may have beneficial effects by not merely removing circulating antibodies, but increasing Treg activity [49, 139–141].

It may be feasible to selectively enhance Treg numbers using a pharmacological approach. The prodrug FTY720 (Fingolimod) is phosphorylated* in vivo* and has been shown to trap naïve and memory T cells in the thymus and secondary lymphoid organs by downregulating sphingosine 1-phosphate receptor 1 (S1P1) [142]. Activation of S1P1 is linked to inhibition of Treg cell differentiation [142]; however, FTY720 and phosphorylated FTY720 increase Treg levels and activity* in vivo* and* in vitro* [143–146]. The exact mechanism is unclear but may require higher doses [144]. The inhibitory effects of atorvastatin on inflammation in acute coronary syndrome (ACS) may be due to its beneficial effects on natural Tregs [147]. In patients with ACS, atorvastatin treatment increased the percentage and inhibitory ability of natural Tregs.

IL-2 plays a critical role in Treg cell activity, growth, and survival, but there may be insufficient IL-2 to sustain their* in vivo* potentiation [12]. Emerging evidence indicates that enhancing Treg cell numbers and activity with low-dose IL-2 treatment is effective in treating autoimmune and inflammatory diseases [12, 148–151], although there may be intrinsic risk as IL-2 is also a key growth factor for effector CD4^+^ T cells and NK cells [12]. An IL-2/anti-IL-2 immune complex is reported to preferentially expand Treg cells with little or no effect on other cells [121]. Delivery of the complex to mice before TAC was recently reported to attenuate LV hypertrophy, inflammation, and contractile dysfunction, while increasing LV levels of Treg cells [94]. Inert biodegradable nanoparticles represent another promising platform for stabilizing and expanding Tregs* in vivo*. McHugh et al. [152] recently reported success in using nanoparticles loaded with the Treg inducers IL-2 and TGF-*β* and targeted to CD4^+^ T cells with conjugated antibodies. These nanoparticles were demonstrated to induce CD4^+^ Tregs* in vitro*, even in the presence of proinflammatory cytokines, and expand their number in mice* in vivo*.

Vitamin D status has been linked to chronic heart failure and vitamin D supplementation improves LV structure and function in heart failure patients [153]. Recent evidence indicates a modulatory role of the vitamin D system in the formation and activity of regulatory T cells [154]. Vitamin D deficiency was associated with reduced numbers and impaired function of naïve CD45RA^+^ regulatory T cell in chronic heart failure patients [120]. In addition, the vitamin D receptor agonist BXL-01-0029 was shown to inhibit IFN-*γ* and TNF-*α*-induced CXCL10 secretion by fetal human cardiac myocytes and reduce CXCL10 protein secretion and gene expression by CD4^+^ T cells [155]. Whether a vitamin D receptor agonist or supplementation increases Treg levels and reduces CXCL10 levels in heart failure patients will need to be assessed.

## 8. Conclusions and Future Perspectives

The last decade has witnessed an impressive advance in our understanding of the role of the immune system in the pathophysiology of cardiovascular diseases. Beyond an expected role in allograft disease and the development and progression of atherosclerosis, an abundant body of evidence supports a significant contribution of the immune system to many other cardiovascular settings, ranging from the development and maintenance of hypertension to cardiac and vessel remodeling (whether constrictive or expansive) in response to hemodynamic stress [156], in both health and disease. The challenge now is to translate this knowledge for the benefit of patients suffering from cardiovascular diseases. Targeting the CXCL10/CXCR3 axis and cardiac inflammation may open new pharmacological venues for treating heart failure or coronary artery disease to supplement current drugs that target the sympathetic or renin-angiotensin systems, or platelets. This will require selectively targeting the most critical immune pathways in order to optimize interference with pathological processes, while preserving protective and homeostatic immune functions. Antigen-specific modulation of the immune system, for example, through systemic delivery of nanoparticles coated with disease-relevant peptides bound to major histocompatibility complex class II (pMHCII) to expand endogenous antigen-specific Tregs [157], is an optimal strategy in settings where an antigen-specific adaptive immune response has been involved in disease development or progression. However, several cardiovascular diseases will probably resist the identification of a specific pathogenic antigen and will require a broader, although targeted, regulation of the immune response, for example, through administration of low-dose IL-2 to promote endogenous Tregs [149]. With regard to CXCR3 pathway, besides the therapeutic possibilities listed above, we believe that the identification and development of selective Evasins [158] or Evasin-like peptides that differentially bind and neutralize CXCL9 or CXCL10 versus CXCL11 may provide an interesting therapeutic strategy to limit pathogenic while preserving regulatory CXCR3 functions.

## Figures and Tables

**Table 1:** Immunological Mediators in Chronic Inflammation of the Heart.

Disease	Elevated Th1	Depressed Tregs	Elevated CXCL10
CAD	1; 27; 31; 32	32–34	35–37
Kawasaki	44	45	46–49
Myocarditis/Chagas	58; 62; 65; 66	62; 65; 67	68; 69; 71
Hypertrophy	83; 84; 86; 92; 93	97–99	84; 89–91
